# The relation between C-reactive protein and serum amyloid A in patients with autoinflammatory diseases

**DOI:** 10.1186/s12969-022-00757-9

**Published:** 2022-11-24

**Authors:** G. E. Legger, C. W. E. Dermer, A. F. Brunger, P. L. A. van Daele, H. L. A. Nienhuis

**Affiliations:** 1 Department of Pediatric Rheumatology, University of Groningen, University Medical Center Groningen, Groningen, The Netherlands; 2 Department of Rheumatology and Clinical Immunology, University of Groningen, University Medical Center Groningen, Groningen, The Netherlands; 3 Department of Internal medicine, section allergy and clinical Immunology, Erasmus University, University Medical Center Rotterdam, Rotterdam, The Netherlands; 4 Department of Internal Medicine, University of Groningen, University Medical Center Groningen, Groningen, The Netherlands

**Keywords:** Amyloidosis, Familial Mediterranean fever, Hereditary autoinflammatory diseases, Inflammation, Acute-phase proteins, C-reactive protein, Serum amyloid A protein

## Abstract

**Background:**

Autoinflammatory diseases are rare disorders of the innate immune system characterized by fever and other signs of inflammation. A feared complication of autoinflammatory diseases is the development of AA amyloidosis. AA amyloidosis is caused by extracellular deposition of soluble serum amyloid A (SAA) proteins as insoluble amyloid fibrils leading to organ damage. Prolonged high levels of SAA are a prerequisite to develop AA amyloidosis. Since measurement of SAA is relatively expensive and sometimes unavailable, C-reactive protein (CRP) is often used as a surrogacy marker to test for inflammation.

**Objective:**

The aim of this research is to evaluate the possible relation between CRP and SAA.

**Methods:**

A retrospective cohort of patients with autoinflammatory diseases (*n* = 99) where SAA and CRP blood testing was performed in the period between 2015 and 2021 in the University Medical Centre in Groningen was used to investigate the correlation between CRP and SAA.

**Results:**

CRP and SAA have a high correlation (rho = 0.755, *p* < 0.001). A CRP value below 0.45 mg/L results in 100% sensitivity for SAA below 4 mg/L. CRP below 5 mg/L is a good predictor of SAA below 4 mg/L with 85.4% sensitivity and 83.6% specificity. Only prednisone and erythrocyte sedimentation rate (ESR) significantly influence the relation between CRP and log_10_*SAA*.

**Conclusion:**

There was a significant correlation between CRP and SAA in our retrospective cohort. CRP levels below 5 mg/L proved to be highly predictive of SAA levels below 4 mg/L. This may not be true for patients on steroids.

## Introduction

Autoinflammatory diseases are a heterogenous group of disorders of the innate immune system. Patients with autoinflammatory diseases usually present with episodes of inflammation, manifesting as fever, increased acute phase proteins in serum, serositis (pleuritis, pericarditis and peritonitis) and/or signs of inflammation in other organs. Recurrent or long-lasting episodes of inflammation may result in organ damage and/or a decreased quality of life. One of the most feared complications is systemic AA amyloidosis. Systemic AA amyloidosis is caused by the accumulation of insoluble amyloid fibrils in organs interfering with the normal physiological functioning of these organs. Since amyloid fibrils in AA amyloidosis are formed by cleavage, misfolding and aggregation of the precursor protein serum amyloid A (SAA), a sustained high concentration of SAA is a prerequisite for developing AA amyloidosis [1–4]. Even if patients with autoinflammatory diseases do not experience any overt clinical symptoms, inflammatory parameters, such as c-reactive protein (CRP) and SAA, can be increased (subclinical inflammation) [2, 5–12]. Although many publications in this field state the importance of regular SAA measurements to screen for inflammation during and between attacks [4, 13], another acute phase reactant, CRP, is often used as a surrogate marker for SAA. Multiple reasons have been named for the use of this surrogate marker. One of them is the impossibility of SAA testing in many parts of the world, another one is that the SAA tests are more expensive than CRP tests [13]. Previous research has been done to evaluate the correlation between CRP and SAA. In these studies, variable correlations between CRP and SAA (rho = 0.2–0.8) [6, 8, 13–20] were obtained. However, study populations across these studies vary greatly. Studies only including patients with Familial Mediterranean Fever (FMF) show a high correlation between CRP and SAA, but they also describe patients with a normal CRP level who did have elevated SAA levels [8, 13], raising the question if there are specific circumstances in which CRP is a less suitable marker to predict SAA values in patients with autoinflammatory diseases. Previous studies already stated that CRP seems to be a less sensitive method for demonstrating subclinical inflammation in patients with FMF in attack free-periods than SAA [7]. We suspect that multiple other factors might influence SAA, as for example medication. Most patients with autoinflammatory diseases are treated with colchicine and/or additional cytokine blocking agents such as Anakinra, Canakinumab (IL-1 blockers), Tocilizumab (IL-6 blocker) or Etanercept (TNF-alpha antagonist) and/or steroids. It is known that improvement or even normalisation of CRP and/or SAA levels can be seen when patients are treated with cytokine blocking agents [4], however, a significant decrease in CRP does not always mean a significant decrease in SAA as illustrated by Wu B. et al. [4, 16]. Another question is: what is the cut-off value for SAA to prevent the development of AA amyloidosis and what is the best cut-off value for CRP to accurately predict a low SAA level. To our knowledge no long term follow up studies have been performed to determine the cut-off value for SAA to prevent the development of AA amyloidosis in auto inflammatory patients. In FMF patients different cut-off values for SAA (4–10 mg/L) are suggested to prevent the development of AA amyloidosis [6, 7, 17]. In AA amyloidosis favourable outcomes in terms of mortality and renal disease progression are seen when SAA levels are below 4 mg/L [4]. In infections and cardiovascular diseases normal values for CRP vary between 1 and 25 mg/L [18, 19]. The best cut-off value for CRP to predict a low SAA level is less clear. In a study with FMF patients, the authors decided upon a threshold value for CRP below 5 mg/L for children and below 8.75 mg/L for adults. This decision was made because a significant correlation between SAA levels and age was discovered. However, in 16% of the patients with a CRP level below these cut-off values, SAA levels were still above 10 mg/L [7]. In line with this, Berkun et al. found that 34% of FMF patients had SAA above 6 mg/L while CRP levels were below a threshold of < 0.5 mg/L [13]. The aim of this study was to establish the relation between CRP and SAA in patients with different autoinflammatory diseases. Furthermore, we wanted to investigate a cut-off value for CRP to accurately predict a normal SAA level and to describe factors that might influence the relation between CRP andserum amyloid A levels in patients with autoinflammatory diseases.

## Methods

This retrospective cohort study was performed in University Medical Centre Groningen (UMCG), the Netherlands. Approval was obtained from the Medical Ethics Committee of the UMCG (2021/050). All subjects, and/or their parents, provided informed consent.

### Study population

Medical records of patients in whom SAA levels were measured between January 2015 and December 2021 were selected for evaluation. Patients were excluded if they did not have an autoinflammatory disease as assessed by the treating physician, or when concomitant CRP measurements were missing.

### Assessments

Clinical characteristics such as sex, age, weight, height, diagnosis, ethnicity, disease activity, presence of infection and usage of medication at the time of blood testing were retrospectively extracted from the medical files. If known, the presence of AA amyloidosis was recorded. Diagnosis of AA amyloidosis was made based on the presence of amyloid fibrils in the abdominal fat or an affected organ. Amyloid deposits had to be characterized histochemically by positive staining with Congo red and typical anomalous colours under polarized light [20]. AA type had to be identified by immunohistochemistry or proteomics [21, 22].

Medication use was recorded solely if prednisone, antibiotics, colchicine, TNF blockers or anti-interleukin medication were taken by the patient. If medication was exclusively taken during attacks, this was recorded separately. Disease activity was based on clinical symptoms of the patient at the moment of the visit conform the autoinflammatory disease activity index (AIDAI) [23]. Patients without symptoms of inflammation were scored as “remission”. Patients with an infection as assessed by the treating physician were scored as having “infection”. Laboratory results for SAA, CRP, ferritin, erythrocyte sedimentation rate (ESR), and leucocytes with differential were extracted.

C-reactive protein (CRP) levels were measured by turbidometry. Serum Amyloid A (SAA) levels were measured using a monoclonal antibody-based sandwich ELISA. Measurement of CRP values below 0.3 mg/L was registered as 0.3, for SAA levels below 1 mg/L the value of 1 was recorded. A CRP level under 5 mg/L was used as normal based on the supposed absence of an acute phase reaction below this level [18]. A SAA level below 4 mg/L was used as normal because of the known favourable outcomes in terms of mortality and renal disease progression in patients with already existing AA amyloidosis [4] and the lack of well-defined cut-off values for SAA values to prevent amyloidosis.

### Statistical analysis

Statistical analyses were performed using IBM Statistical Packages for Social Sciences (SPSS), version 23. A *p*-value of below 0.05 was considered statistically significant. Descriptive statistics were described as number (%) of patients for categorical parameters and mean (standard deviation; SD) or median (interquartile range; IQR) for normally or non-normally distributed continuous parameters. Kolmogorov-Smirnov as well as visual methods (histograms and probability plots) were used to check for normal distribution. Spearman correlation tests were performed between CRP, ESR, number of granulocytes or lymphocytes, ferritin level, BMI, age and SAA in all visits. A ROC curve was constructed to evaluate the best cut-off for CRP to accurately predict a SAA below 4 mg/L. A crosstab was created to measure the sensitivity and specificity of a CRP level under 5 mg/L to predict a SAA level under 4 mg/L and to predict a SAA level under 10 mg/L. False positive and negative and true positive and negative predictions were described in detail. False negative predictions were compared with true negative predictions and false positive predictions with true positive predictions by independent samples t-test. Separate Spearman correlation tests were performed between CRP and SAA only in the first visits of a patients not using prednisone versus the first visits of patients using prednisone and in the groups based on disease activity state (remission, flare, infection). (Multivariate) linear regression analyses were performed to detect the (independent) relation between different (inflammatory) parameters and SAA. A log_10_ transformation of SAA values was performed to increase the homogeneity of variance.

## Results

In total, 1099 patients with a total of 7734 measurements of SAA were registered in a sequential database. After exclusion of patients without an autoinflammatory disease and SAA measurements without concomitant CRP measurement a total of 99 individual patients with a total of 381 combined measurements were included. Patients characteristics are shown in Table 1.

**Table 1:** Patient characteristics

Total number of patients (n)	99
Combined SAA and CRP measurements (n)	381
Number of visits with combined measurement per patient, median (minimum-maximum)	3 (1–13)
Visits while on specific medication, n (%)	Prednisone 28 (7.3%), Anakinra 59 (15.5%), Canakinumab 67 (17.6%), Tocilizumab 6 (1.6%), Colchicine 166 (43.6%), Humira 3 (0.8%), antibiotics 4 (1%)
Male sex, n (%)	56 (57%)
Age (years), median (interquartile range)	21 (11–38)
BMI (kg/m2), mean (±SD)	22.53 (±5.75)
Autoinflammatory diagnoses (n)	FMF (45), CAPS (9), sJIA (5), Schnitzler syndrome (4), TRAPS (3), hidradenitis suppurativa (3), adult onset still’s disease (AOSD) (3), Behcet disease (2), PFAPA (2), MKD (2), PAPA (1), DADA2 (1) and unclassified autoinflammatory diagnosis (19)
AA amyloidosis present, n (%)	Yes 11 (11.1%), No 88 (88.9%%)
Ethnicity, n (%)	Caucasian 45 (45.9%), Arabic 33 (33.7%), Armenian 13 (13.1%), Asian 2 (2%), Afro-American 1 (1%), Russian 1 (1%), Georgian 1 (1%), unknown 3 (2%).

Abbreviations: *SAA* Serum amyloid A, *CRP* C-reactive protein, *BMI* Body mass index, *FMF* Familial Mediterranean Fever, *sJIA* Systemic onset juvenile idiopathic arthritis, *TRAPS* Tumor necrosis factor receptor associated periodic syndrome, *MKD* Mevalonate kinase deficiency, *PAPA* Pyogenic arthritis pyoderma gangrenosum and acne, *DADA2* Deficiency of adenosine deaminase 2

**Fig. 1:**
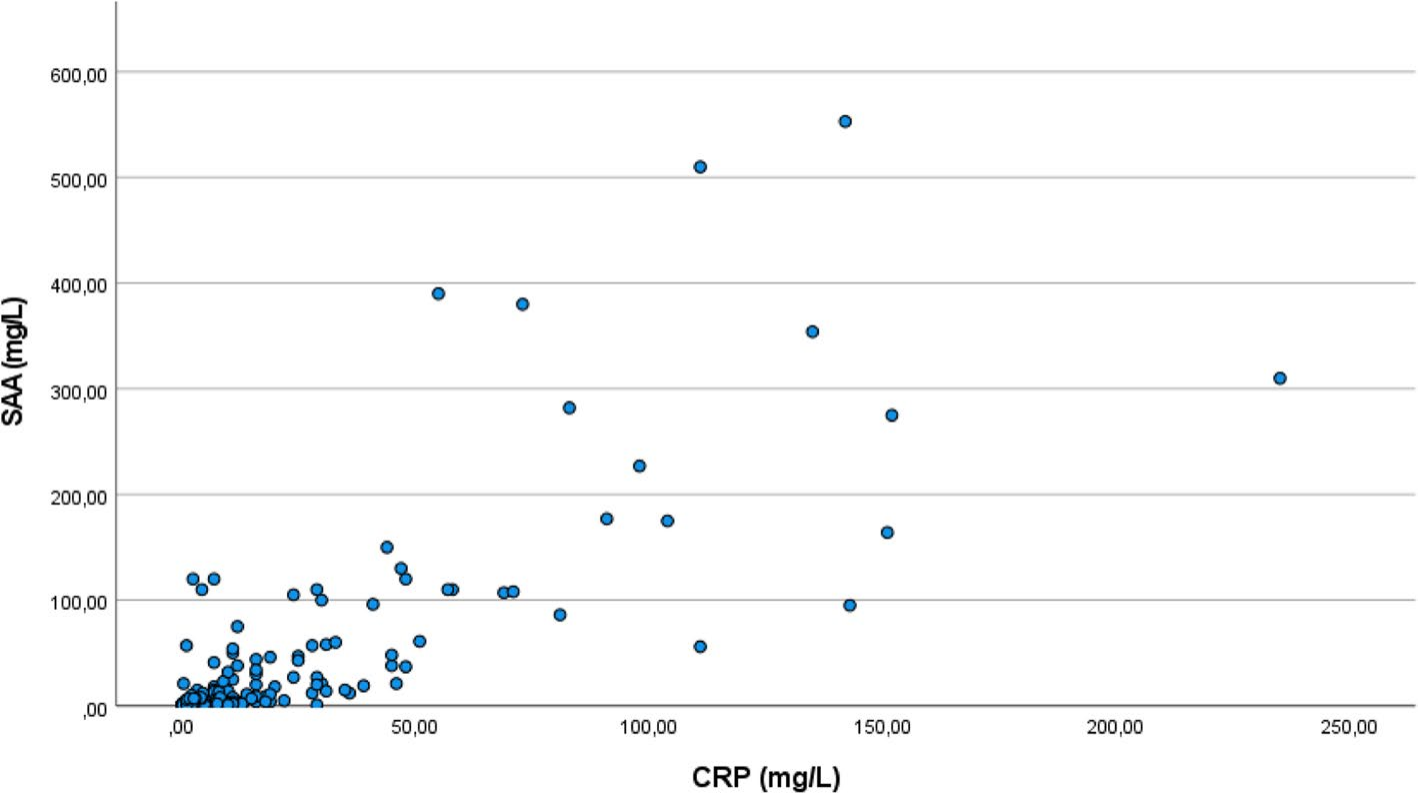
Scatterplot of all Serum Amyloid A and C-reactive protein values

The median value of CRP measurements was 2 mg/L (IQR; 0.4–9.0), with a minimum of 0.3 mg/L and a maximum of 235 mg/L. The median value of SAA measurements was 1 mg/L (IQR; 0.5–4.5), with a minimum of 0.5 mg/L and a maximum of 553 mg/L. Neither CRP nor SAA levels were normally distributed (Kolmogorov-Smirnov test, both *p* < 0.001). Figure 1 shows a scatterplot of all CRP and SAA levels.

When including all measurements, CRP correlated strongly with SAA levels (rho = 0.755, *p* < 0.001). When including only data from the first visits, CRP also strongly correlated with SAA levels (rho = 0.803, *p* < 0.001). Because there were no big differences between the results using all visits compared to only the result of the first visits we decided to include all visits for further analysis. Calculation of the correlation coefficient between different parameters and SAA or CRP gave results as described in Table 2. The highest correlation was seen between CRP and SAA (rho = 0.755, *p* < 0.001). Looking at the correlations in different ethnic groups we showed that the correlation between CRP and SAA was higher in the Caucasian group than in the Arabic and Armenian group (rho = 0.873, *p* < 0.001 versus rho = 0.771, *p* < 0.001 and rho = 0.778, *p* < 0.001). When comparing the correlation between lymphocytes and SAA separately for the visits with and without prednisone, the correlation was significant but low in the group with prednisone and very low and not significant in the group without prednisone (rho = − 0.565, *p* = 0.015 versus rho = − 0.124, *p* = 0.039).

**Table 2:** Spearman correlation analysis of different markers and SAA and CRP in all visits

	SAA (rho=)	Significance (***p***-value)	CRP (rho=)	Significance (***p***-value)	N
CRP (mg/L)	0.755	< 0.001*	1	–	381
ESR (mm/h)	0.550	< 0.001*	0.620	< 0.001*	344
Granulocytes (n)	0.291	< 0.001*	0.396	< 0.001*	297
Ferritin (ug/L)	0.327	0.002*	0.491	< 0.001*	85
Lymphocytes (n)	−0.173	0.003*	−0.161	0.006*	296
BMI (kg/m2)	0.255	0.017*	0.343	0.001*	87
Age (years)	0.221	< 0.001*	0.404	< 0.001*	380

*Abbreviations*: *CRP* C-reactive protein, *ESR* Erythrocyte sedimentation rate, *BMI* Body mass index

* Indicates significant *p*-value

**Fig. 2:**
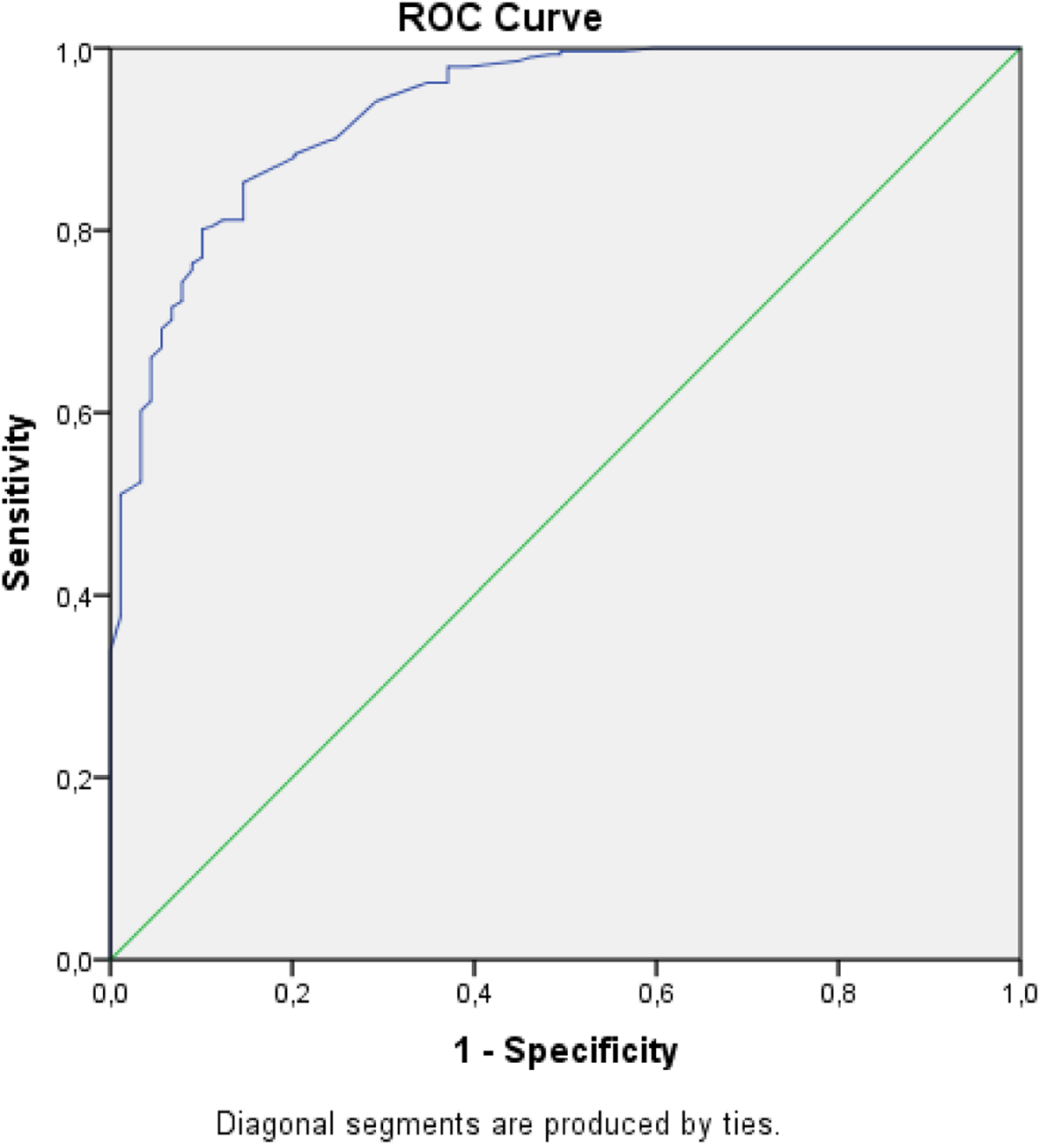
Receiver Operating Characteristic curve: CRP values for SAA

For all measurements of CRP and SAA the ROC curve as presented in Fig. 2 resulted in an area under the curve of 0.932 with a maximum CRP value of 0.45 mg/L keeping a 100% sensitivity and a 33.9% specificity level that the SAA level is below 4 mg/L.

To evaluate whether a CRP level < 5 mg/L accurately predicted a SAA value < 4 mg/L and < 10 mg/L we cross tabulated them (Table 3). Sensitivity of CRP < 5 mg/L to predict SAA levels < 4 mg/l was 85.4% with a specificity of 83.6% with a positive predictive value of 61% and a negative predictive value of 95%. The sensitivity of CRP < 5 mg/L to predict SAA levels < 10 mg/L was 91.9% with a specificity of 81.8% with a positive predictive value of 55% and a negative predictive value of 81%.

**Table 3:** Crosstab CRP and SAA above clinically relevant values

		SAA above 4 mg/L			SAA above 10 mg/L	
		Yes	No		Yes	No
**CRP above 5 mg/L**	**Yes**	76 (85.4%)	48 (16.4%)	124 (32.5%)	68 (91.9%)	56 (18.2%)
	**No**	13 (14.6%)	244 (83.6%)	257 (67.5%)	6 (8.1%)	251 (81.8%)
**Total**		89 (23.3%)	292 (76.7%)	381	74 (19.4%)	307 (80.6%)

*Abbreviations*: *CRP* C-reactive protein, *SAA* Serum amyloid A

In total, 244 true negative (64%), 13 false negative (3.4%), 76 true positive (19.9%) and 48 false positive (12.6%) predictions were found in the 381 visits in 99 patients (Table 4). Certain differences in the populations can be observed when comparing the false positives with the true positives and the false negatives with the true negatives. The age seems to be higher in the false positive group but this is not significant. The BMI of the false positive group is significantly higher than the BMI of the true positive group (*p* < 0.001). Some differences in medication use were observed. For example, Colchicine usage seems to be lower in the false negative group compared to true negatives (30.8% versus 47.1%), Canakinumab usage seems to lower in the false negative group compared to the true negatives (7.7% versus 20.1%)

**Table 4:** Description of the true and false positive and true false negative measurements

	All measurements	True positives	False positives	True negatives	False negatives
Number of patients (n)	99				
Total number of visits (n)	381	76	48	244	13
Age (years) median (IQR)	21 (11–38)	23 (10–35)	36 (23–57) (*p* = 0.240)	18 (9–30)	17 (15–58) (*p* = 0.531)
BMI (kg/m2), mean (±SD)	22.53 (±5.75)	21.96 (±4.56)	28.16 (±7.25) (*p* < 0.001)*	21.80 (±5.60)	23.77 (±0.34) (*p* = 0.875)
CRP values mg/L, median (IQR)	2.00 (0.40–9.00)	29 (12.00–54.00)	10 (7.00–11.00)	0.70 (0.30–1.80)	2.70 (1.40–4.25)
SAA values mg/L, median (IQR)	1.00 (0.50–4.50)	43.50 (15.75–109.50)	2.00 (1.00–4.00)	1.00 (1.00–1.00)	10.00 (7.00–39.00)
Visits with medication usage, n (%)	Colchicine 166 (43.6%),	Colchicine 27 (35.5%),	Colchicine 20 (41.7%),	Colchicine 115 (47.1%),	Colchicine 4 (30.8%),
	Canakinumab 67 (17.6%),	Canakinumab 8 (10.5%),	Canakinumab 9 (18.8%),	Canakinumab 49 (20.1%),	Canakinumab 1 (7.7%),
	Anakinra 59 (15.5%),	Anakinra 16 (21.1%),	Anakinra 8 (16.6%),	Anakinra 33 (13.5%),	Anakinra 2 (15.4%),
	Prednisone 28 (7.3%),	Prednisone 11 (14.5%),	Prednisone 1 (2.1%),	Prednisone 13 (5.3%),	Prednisone 3 (23.1%),
	Tocilizumab 6 (1.6%),	Tocilizumab 0, (0%)	Tocilizumab 0, (0%)	Tocilizumab 6 (2.5%), (0%)	Tocilizumab 0, (0%)
	Humira 3 (0.8%),	Humira 0, (0%)	Humira 0, (0%)	Humira 3 (1.2%),	Humira 0, (0%)
	antibiotics 4 (1%),	antibiotics 1 (1.3%),	antibiotics 2 (4.2%),	antibiotics 1 (0.4%),	antibiotics 0, (0%)
	no medication 48 (12.6%)	no medication 13 (17.1%)	no medication 0 (0%)	no medication 24 (9.8%)	no medication 4 (30.8%)
Conclusion of visit based on expert opinion and ADAI, n (%)	Flare up AID 40 (10%),	Flare up AID 22 (28.9%),	Flare up AID 7 (15%),	Flare up AID 11 (4.5%),	remission 13 (100%)
	remission 320 (84%),	remission 45 (59.2%),	remission 37 (77%),	Remission 225 (92.6%),	
	infection 8 (2%)	infection 4 (5.3%),	infection 2 (4%),	Infection 2 (0.8%),	
	unknown 13 (3%)	Unknown 5 (6.6%)	Unknown 2 (4%)	unknown 6 (2.5%)	

*Abbreviations*: *IQR* Interquartile range, *BMI* Body mass index, *AID* Autoinflammatory disease, *CRP C*-reactive protein, *ESR* Erythrocyte sedimentation rate, *ADAI* Autoinflammatory disease activity index

*Indicates a significant *p*-value between the false and the true groups

Prednisone usage seems to be lower in the group of false positives compared to true positives (2.1% versus 14.5%) but higher in the false negatives compared to the true negatives (23.1% versus 5.3%). The percentage of patients in remission seems to be higher in the false positive group compared to the true positives (77% versus 59.2%). All patients were in remission during the visits in the false negative group compared to 92% of the true negatives.

The correlations at the first visits with (*n* = 12) and without prednisone (*n* = 92) were analysed. The correlation was significant in both groups and is slightly lower at the first visits with prednisone (rho = 0.771 *p* = 0.003 versus rho = 0.824, *p* < 0.001). Median SAA/CRP ratios at the visits with prednisone seem to be slightly higher compared to the visits without prednisone (median = 1.04, IQR 1.04–3.03 versus median = 1, IQR 0.44–3.33). Correlations between CRP and SAA at visits with an autoinflammatory disease flare, disease remission and an infection were analysed separately. The correlation was significant in all groups and is higher during flare up of the autoinflammatory disease than during remission and infection (rho = 0.911, *p* < 0.001 versus rho = 0.690, *p* < 0.001 and rho = 0.795, *p* = 0.0018).

Univariate linear regression analysis revealed a significant association between CRP and log_10_*SAA* (B = 0.233, 95% CI 0.181;0.284, *p* < 0.001, explained variance 54.5%). Other significant predictors of log_10_SAA were age, ESR, lymphocytes, granulocytes, ferritin levels and prednisone use. However, only ESR and prednisone contributed significantly to the multivariate model with CRP (Table 5).

**Table 5:** Univariate and multivariate linear regression analyses with log_10_SAA

	Univariate B (95% CI)	***p***-value	Multivarariate B (95% CI)	***p***-value
CRP (mg/L)	0.020 (0.018; 0.022)	< 0.001*	0.017 (0.015; 0.019)	< 0.001
Age (years)	0.006 (0.002; 0.009)	0.002*	–	n.s.
BMI (kg/m2)	0.007 (−0.021; 0.035)	0.671		
Sex (m/f)	0.020 (−0.284; 0.324)	0.896		
AA amyloidosis (no/yes)	0.032 (−0.241; 0.034)	0.891		
ESR (mm/h)	0.015 (0.013; 0.018)	< 0.001*	0.005 (0.002; 0.007)	< 0.001
Lymphocytes (10^9/l)	−0.184 (− 0.228; − 0.068)	< 0.001*	–	n.s.
Granulocytes (10^9/l)	9.119 (5.253; 12.985)	< 0.001*	–	n.s.
Ferritin (ug/L)	0.005 (0.003; 0.007)	< 0.001*	–	n.s.
Antibiotics (no/yes)	0.282 (−0.394; 0.958)	0.413		
Prednisone (no/yes)	0.416 (0.150; 0.683)	0.002*	0.232 (0.049; 0.415)	0.013
Anakinra (no/yes)	0.143 (−0.051; 0.338)	0.149		
Canakinumab (no/yes)	−0.168 (− 0.353; 0.016)	0.074		
Colchicine (no/yes)	−0.074 (− 0.217; 0.068)	0.306		
Medication taken during attack (no/yes)	−0.163(− 0.534;0.208)	0.387		

*Abbreviations*: *CRP* C-reactive protein, *SAA* Serum amyloid A, *ESR* Erythrocyte sedimentation rate

*indicates significant *p*-value, *n.s* Not significant

## Discussion

The association between CRP and SAA was high in our population (rho = 0.755, *p* < 0.001) (Table 2), in accordance with other studies done in similar specific populations: patients with FMF [24], patients with hidradenitis suppurativa [25] and patients with infectious diseases [14]. The association between CRP and SAA was significant across the ethnicities in this study, but seems to be a little higher in the Caucasian population compared to the Armenian and Arabic population. In most studies we know of in this field, a minority of patients were Caucasian, since most studies were done in FMF patients and most of the FMF patients are not Caucasian. The difference in association across ethnicities might be explained by more or less prevalent diseases within ethnic groups. Other markers with a significant positive correlation with SAA were ESR, ferritin and number of granulocytes. This may be explained by the fact that these parameters increase during activation of the innate immune system, similar to SAA. The association between ESR and SAA that we found (rho = 0.550, *p* < 0.001) is in agreement with previous research [24]. The number of lymphocytes was weakly inversely associated with SAA (rho = − 0.173, *p* = 0.003). These finding may be explained by the fact that in autoinflammatory diseases the innate immune system is more active than the adaptive immune system. Although significant, the association between age and BMI with SAA in this study was very weak (rho = 0.221, *p* < 0.001; rho = 0.255, *p* = 0.017). This positive association between age and BMI with acute phase reactants is also observed in other populations [7]. With advancing age chronic antigen stimulation and accumulation of cellular residues affects both the innate and the adaptive part of the immune system. For the innate immune system the consequence is inflammaging (low grade clinically undetectable inflammation with increased production of pro-inflammatory cytokines) and for the adaptive immune system it is immunosenescence (immune dysfunction that progresses with age). Inflammaging is considered as a basis of most age-related diseases and might explain the weak correlation between age and SAA levels [7, 26]. The association between BMI scores and SAA levels can be explained by the widely accepted relation between adiposity and inflammation, through IL-6 secretion by adipose tissue [27].

Based on our study, if a 100% sensitivity of SAA levels below 4 mg/L is desired, a CRP cut-off value of 0.45 mg/L should be used (Fig. 2). The best CRP cut-off value to detect low SAA levels is still a discussion: chosen values range from 0.5–10 mg/L, largely depending on the desired sensitivity and specificity [4, 7, 13, 14]. In our opinion further decrementation of CRP levels below the value 5 mg/L is not clinically relevant as CRP levels under 5 mg/L are used as normal based on the supposed absence of an acute phase reaction below this level [18]. When using the predetermined cut-off value of 5 mg/L for CRP, 85.4% sensitivity and 83.6% specificity were found to predict a SAA level < 4 mg/L, which in our opinion are acceptable values for use day to day. The sensitivity and specificity of a cut-off value for CRP changes when different levels of SAA are pursued. In relevant literature different cut-off values for SAA are used. Choices are based on possible damage to organs and/or increased risk of AA amyloidosis and vary between 4 and 10 mg/L [4, 7, 13, 14]. We have chosen a cut-off value of SAA below 4 mg/L, because of known favourable outcomes in terms of mortality and renal disease progression in patients with already existing AA amyloidosis [4]. However, the best cut-off value is still debatable.

When comparing the false positives to the true positives and the false negatives to the true negatives, the number of patients in remission was higher in the false positive group compared to the true positives (77% versus 59.2%) and all patients were in remission during the visits in the false negative group compared to 92% of the true negatives. The correlation between CRP and SAA was lower in the group in remission compared to the group with a flare (rho = 0.690 versus rho = 0.911). These results are in line with other research in FMF patients where CRP seems to be a less suitable marker to predict elevated SAA levels in FMF patients in an attack-free period. The frequency of colchicine use was lower in the false negative group compared to true negatives (30.8% versus 47.1%) and slightly higher in the false positive group compared to the true positive group (41.7% versus 35.5%). Colchicine decreases or even inhibits the production of SAA, as reported in mice studies [13, 28]. The higher frequency of colchicine use in the false positive group and lower frequency of colchicine use in the false negative group might be explained by the assumption that SAA levels are more influenced by colchicine than the CRP levels.

The frequency of prednisone use was higher in the false negative group compared to the true negatives (23.1% versus 5.3%)Age, BMI, use of medication and disease activity state may vary over time in the included patients. Therefore, we decided to include all available combined SAA and CRP measurements for all patients in the analysis shown in Table 4. But some patients have more frequent visits than other and as a consequence, their results may have weighed more than the results of other patients, thus resulting in a bias. For this reason, we also looked at the correlation between SAA and CRP and the SAA/CRP ratios, when only looking at the first visits of patients with and without prednisone. The correlation between CRP and SAA seems to be lower and the SAA/CRP ratio seems to be higher in the group with prednisone compared to the group without prednisone. Apart from that, the multivariate linear regression analysis revealed CRP, prednisone usage and ESR as independent predictors of log_10_*SAA* (Table 5). The function of prednisone, and other corticoids, in relation to inflammation is still not fully understood. Glucocorticoids seem to have both suppressing and stimulatory effects on the immune system, depending on the concentration of glucocorticoids in the body [29]. A study in healthy canines describes non-significant changes in CRP and SAA levels after prednisone administration [30]. No other research has looked into the effect of prednisone on acute phase reactants in humans to the best of our knowledge. Our results suggest that clinicians should be careful with interpretation of CRP levels to predict SAA levels in patients using prednisone.

The association of increased ESR levels with SAA might be the result of the presence of more proteins in the blood increasing the erythrocyte sedimentation rate. All of this should be taken into account by physicians interpreting CRP values to predict SAA levels. If a patient shows elevated ESR levels and/or uses prednisone it is likely that increased SAA levels are present, even with a low CRP.

A limitation of this retrospective study design is that, besides CRP and SAA, various other markers for inflammation (such as interleukin-18, interleukin-6, ferritin, and body temperature) were not investigated. Another limitation of this study is that we didn’t gather information about the exact time point of the blood test in time relation to the disease episode. Previous research showed that, although CRP and SAA are both sensitive biomarkers for inflammation in the early phase of an inflammatory disease, the kinetics of CRP and SAA levels herein are not completely similar. The half-life times of SAA levels are significantly shorter than that of CRP [31] and this will influence the correlation between SAA and CRP levels at different time points throughout the disease period.

Another limitation of this study was that we did not include genetic test results in the analysis of the relation between CRP and SAA since this might also play a role [7]. Furthermore, we included a relative small population (*n *= 99), with a larger variety of autoinflammatory diseases, making it a very heterogenous group. These limitations should be taken into account when interpretating the results of this study.

## Conclusions

We found a high correlation between C-reactive protein and serum amyloid A in a population of patients with autoinflammatory diseases (rho = 0.755, *p* < 0.001). CRP levels below 5 mg/L proved to be highly predictive of SAA levels below 4 mg/L (sensitivity 85.4% and specificity 83.6%). At a threshold CRP value of 0.45 mg/L, there was even a 100% sensitivity for a SAA levels below 4 mg/L. However, clinicians should be aware that raised ESR and steroid use may be confounding variables for this correlation in patients. We propose that serial CRP measurements, without additional SAA requests, are a cost-effective way to monitor disease activity in patients with autoinflammatory diseases, but not for patients on steroids.

## Data Availability

The datasets used and/or analyzed during the current study are available from the corresponding author on reasonable request.

## Abbreviations

SAA: Serum amyloid A
CRP: c-reactive protein
FMF: Familial Mediterranean Fever
UMCG: University Medical Centre Groningen
BMI: Body mass index
sJIA: Systemic onset juvenile idiopathic arthritis
TRAPS: Tumor necrosis factor receptor associated periodic syndrome
MKD: Mevalonate kinase deficiency
PAPA: Pyogenic arthritis pyoderma gangrenosum and acne
DADA2: Deficiency of adenosine deaminase 2
